# Wnt/beta-catenin pathway: modulating anticancer immune response

**DOI:** 10.1186/s13045-017-0471-6

**Published:** 2017-05-05

**Authors:** Sachin Gopalkrishna Pai, Benedito A. Carneiro, Jose Mauricio Mota, Ricardo Costa, Caio Abner Leite, Romualdo Barroso-Sousa, Jason Benjamin Kaplan, Young Kwang Chae, Francis Joseph Giles

**Affiliations:** 1 Developmental Therapeutics Program, Division of Hematology/Oncology, Feinberg School of Medicine, Chicago, IL USA; 2 Robert H. Lurie Comprehensive Cancer Center of Northwestern University, Chicago, IL USA; 3 Current Address: Department of Interdisciplinary Clinical Oncology, Mitchell Cancer Institute, University of South Alabama, 1660 Springhill Avenue, Mobile, AL USA; 4 Instituto do Câncer do Estado de São Paulo, University of São Paulo, São Paulo, Brazil; 5 A.C. Camargo Cancer Center, São Paulo, Brazil; 6 Department of Medical Oncology, Dana-Farber Cancer Institute, Boston, MA USA

**Keywords:** Wnt, β-catenin, Immunotherapy, Cancer immune regulation, Immune exclusion

## Abstract

Wnt/β-catenin signaling, a highly conserved pathway through evolution, regulates key cellular functions including proliferation, differentiation, migration, genetic stability, apoptosis, and stem cell renewal. The Wnt pathway mediates biological processes by a canonical or noncanonical pathway, depending on the involvement of β-catenin in signal transduction. β-catenin is a core component of the cadherin protein complex, whose stabilization is essential for the activation of Wnt/β-catenin signaling. As multiple aberrations in this pathway occur in numerous cancers, WNT-directed therapy represents an area of significant developmental therapeutics focus. The recently described role of Wnt/β-catenin pathway in regulating immune cell infiltration of the tumor microenvironment renewed the interest, given its potential impact on responses to immunotherapy treatments. This article summarizes the role of Wnt/β-catenin pathway in cancer and ongoing therapeutic strategies involving this pathway.

## Background

Nusse and Varmus discovered the components of the Wnt/β-catenin pathway in 1982 while studying oncogenic mechanisms of mouse mammary tumor virus (MMTV) [1]. Proviral insertion at the “integration site” was thought to be the mechanism of carcinogenesis, giving the name to the first gene discovered in this pathway as *INT1.* Simultaneous work in developmental biology and work in drosophila established *INT1* gene to be the homologue of the Drosophila segment polarity gene, Wingless [2]. Subsequently, human *INT1* was shown to be very similar to mouse *INT1*, thereby revealing the highly conserved nature of this pathway across various species [3]. Additional screens for MMTV proviral insertion sites in tumors yielded several other activated genes that are related to other developmental gene pathways, such as *INT2*, *INT3*, and *INT4* [4–6]. For example, *INT2* is a member of the fibroblast growth factor (FGF; INT2 is the same FGF-3 protein) family, and *INT3* is related to the NOTCH gene family (INT3 protein is the same neurogenic locus notch homologue 4/NOTCH4) [7, 8]. With “INT” nomenclature turning out to be inadequate and confusing, consensus was reached to create the hybrid name “WNT” (for Wingless-related integration site) to denote genes belonging to the INT1/Wingless family. *INT1*—now called *WNT1*—became the founding member [9].

**Fig. 1:**
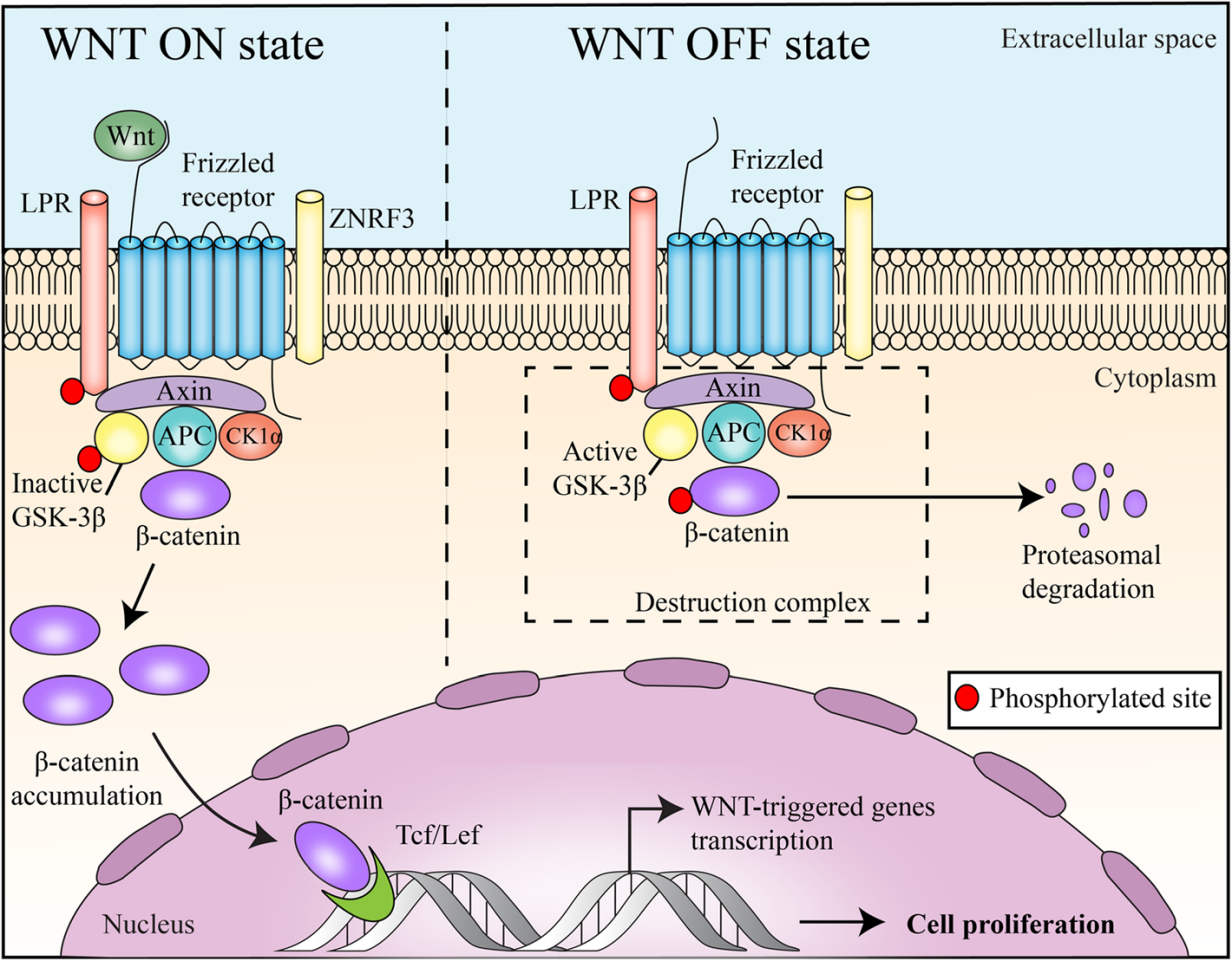
Canonical Wnt/β-catenin pathway: “WNT ON state”: WNT proteins, by binding to frizzled receptors and the LRP co-receptor, act to suppress the activity of glycogen synthase kinase-3β (GSK-3β). ZNRF3 promotes degradation of WNT receptor functioning as tumor suppressors. This prevents phosphorylation of downstream molecules allowing β-catenin association with Tcf/Lef in the nucleus and subsequent increased cell proliferation. “WNT OFF state”: In the absence of WNT ligand, the destruction complex of β-catenin (marked by dotted line box), a tertiary complex formed by axin, APC, CK1α and GSK 3β, will phosphorylate β-catenin, which subsequently undergoes proteasomal degradation

WNTs (translated products of *WNT* gene) are cysteine-rich glycoproteins, secreted by cells into the extracellular matrix, that activate receptor-mediated signaling with cells in immediate proximity [10]. The WNT protein family consists of at least 19 secreted glycoproteins (350–400 amino acids in length) highly conserved across species from invertebrates to mammals [11]. WNT binds to the N-terminal extra-cellular cysteine-rich domain of a Frizzled family receptor, a member of the superfamily of G-protein-coupled receptors. This disrupts the destruction complex of β-catenin (a tertiary complex formed by axin, adenomatous polyposis coli (APC), CK1α, and GSK3β) and triggers the cytoplasmic accumulation of β-catenin (Fig. 1).

T cell factor/lymphoid enhancer factor-1 (TCF/Lef1) is the transcription complex that mediates canonical WNT-triggered gene transcription [12, 13]. β-catenin translocates into the nucleus where it interacts with TCF/Lef1 and activates TCF/Lef1 transcription complex [14–16]. β-catenin also localizes to multiple subcellular locations including the cytoplasm where its levels are tightly controlled. β-catenin also promotes cell-to-cell adhesion by accumulating in cell–cell contact sites, namely the adherens junctions [17, 18]. Figure 1 illustrates the canonical pathway of Wnt/β-catenin signaling. In addition to classical canonical WNT-induced activation of β-catenin–TCF/Lef1 transcriptional complexes, WNT can elicit alternative responses through β-catenin independent mechanisms which are collectively known as noncanonical pathways [19]. In an alternative concept known as integrated Wnt pathway, the canonical and noncanonical pathways are combined and multiple inputs at the level of both Wnt-receptor binding and the downstream, intracellular response have been integrated [20].

Wnt/β-catenin pathway is intricately involved in pathogenesis of several cancers. Recent findings of its role in regulating immunomodulation have renewed enthusiasm in the field.

## Wnt/β-catenin pathway involvement in several malignancies

### Colorectal cancers (CRCs)

The role of the Wnt/β-catenin pathway in carcinogenesis was first described in the setting of *APC* gene mutation. *APC* mutations, which typically are acquired early in the pathogenesis of most colon cancers (over 80%), lead to cytosolic accumulation of β-catenin that in combination with TCF/Lef1 shuttles to the nucleus where it functions as a transcription factor and promotes cellular proliferation [21, 22]. Nuclear expression of β-catenin has been associated with more aggressive cancer biology. In one study, nuclear expression of β-catenin was present in 18 out of 25 (72%) cases of ulcerative CRC while present in only 7 out of 26 (26.9%) cases of polypoid CRC (*P* < 0.001). This finding was independent of APC mutation and E-cadherin expression [23]. The Wnt/β-catenin pathway is also involved in cross talk with the Hippo/YAP pathway. Konsavage et al. showed that β-catenin/TCF4 complexes bind a DNA enhancer element within the first intron of the *YAP* gene to drive YAP expression in CRC cells, contributing to carcinogenesis [24]. The Hippo–YAP signaling pathway could be an effector pathway downstream from APC, independent from its involvement in the β-catenin destruction complex as well [25].

The WNT pathway has been implicated in the maintenance of cancer stem cells (CSC) in colorectal cancer. In vitro data suggest that chronic chemotherapeutic stress-induced “stemness” is associated with attenuated WNT signaling [26]. Vermeulen et al. showed that high activity of the WNT pathway was observed preferentially in tumor cells located close to stromal myofibroblasts, which are thought to secrete factors such as hepatocyte growth factor (HGF) that activate β-catenin-dependent transcription. This maintains CSC clonogenicity and restores the CSC phenotype in more differentiated tumor cells both in vitro and in vivo [27]. HGF additionally induces β-catenin nuclear translocation through Met/β-catenin dissociation, in a Wnt-independent pathway [28].

### Noncolorectal gastrointestinal cancers

β-catenin mutations have been implicated in early steps of carcinogenesis by activating the WNT pathway in gastric and non-hepatitis-related hepatocellular cancers [29, 30]. Cholangiocarcinoma has also been shown to be a WNT-dependent cancer, which thrives on canonical WNT pathway upregulation. Activation of Wnt/β-catenin signaling, by the pluripotent mesenchymal stem cells recruited to the tumor site, seems to play a central role in tumor microenvironment modulation by promoting metastatic growth and resistance to chemotherapy in cholangiocarcinoma [31]. M2-polarized tumor-associated macrophages (TAMs) in the surrounding stroma are known to maintain a highly activated WNT pathway in the tumor [32].

### Desmoid tumors

Crago et al. demonstrated that mutations affecting *APC* and *CTNNB1* (gene encoding β-catenin) occur frequently in desmoid tumors (111 of 117; 95%). Even true *CTNNB1* wild-type tumors (determined by next-generation sequencing) may have genomic alterations associated with WNT activation such as chromosome 6-loss/*BMI1* mutation, supporting Wnt/β-catenin activation as an important pathway governing desmoid initiation [33].

### Breast cancer

WNT/β-catenin signaling regulates the self-renewal and migration of CSCs, thereby promoting tumor growth and metastasis in breast cancer [34]. TAMs in the invasive front of primary mammary tumors have higher expression of molecules involved in WNT-signaling suggesting a role in tumor cell migration, invasiveness, and metastasis [35]. In triple negative breast cancers, β-catenin expression was associated with poor overall survival and disease-specific survival [36]. Upregulation of Wnt/β-catenin has been shown to be a mechanism of resistance to PI3K inhibitors, and the use of β-catenin inhibitors may sensitize PIK3CA mutant breast cancer to PI3K inhibition [37].

### Adrenocortical tumors

Wnt/β-catenin activation has been shown to be an independent prognostic factor of overall and disease-free survival in patients with resected primary adrenocortical cancer. The presence of β-catenin nuclear staining was significantly associated with higher tumor stage and risk score, frequent necrosis, mitoses, and other associated mutations [38]. Results from animal models have suggested that β-catenin can function as an adrenal oncogene causing progressive sub-capsular cell hyperplasia, ectopic expansion of spongiocytes and sub-capsular cell with resulting dysplasia, and marked differentiation defects resulting in primary hyperaldosteronism and development of malignant characteristics such as uncontrolled neovascularization and local invasion when there is prolonged activation [39].

### Melanoma

Wnt/β-catenin activation may have conflicting roles in the metastatic spread of melanoma [40]. β-catenin signaling decreased the migration of melanocytes and melanoma cell lines in vitro but promoted lung metastases in the NRAS-driven melanoma murine model. β-catenin seems to be a major driver of melanoma dissemination to lymph nodes and lungs in a mouse model based on melanocyte-specific *PTEN* loss and *BRAF* (V600E) mutation [41]. β-catenin level also controls tumor differentiation and regulates both MAPK/Erk and PI3K/Akt signaling. In fact, activation of Wnt/β-catenin and AKT pathways mediates chemo-resistance and increased invasion in melanoma cell lines [42].

### Glioblastoma multiforme

Components of WNT pathway are usually overexpressed in glioblastoma multiforme (GBM) tumors. PLAG2 overexpression may play a role in inducing upregulation of WNT6, FZD9, and FZD2, ultimately leading to the maintenance of stemness features of GBM stem cells [43]. β-catenin also increases the expression DNA repair enzyme O6-methylguanine-DNA methyltransferase (MGMT) through Tcf/Lef binding located in the hmMGMT 5′-flanking regulatory region. Genetic or pharmacological inhibition of Wnt/β-catenin signaling reduces MGMT expression and increases the cytotoxic effects of temozolomide [44].

### Renal cell carcinoma (RCC)

Higher levels of β-catenin are associated with poor prognosis, higher stage, node involvement, vascular invasion, and sarcomatoid differentiation in RCC [45]. Multilayer-omics analysis including methylome and transcriptome analyses also demonstrated a significant role of Wnt/β-catenin signaling pathway in RCC pathogenesis [46]. Mutations in *GCN1L1*, *MED12*, and *CCNC*, members of the CDK8 mediator complex that directly regulate β-catenin-driven transcription, were identified in 16% of the RCCs [46].

### Osteosarcoma

Wnt receptor LRP-5 expression correlates with a worse event-free survival in patients with osteosarcoma [47]. Targeting LRP5 receptor signaling with a dominant-negative form of the receptor inhibited tumor growth and metastasis and reduced the expression of cancer cell invasiveness-associated markers in animal models of osteosarcoma.

### Hematological malignancies

Wnt/β-catenin signaling pathway is required for self-renewal of CSCs. Yeung et al. demonstrated that β-catenin was activated during development of mixed-lineage leukemia (MLL) CSCs. Suppression of β-catenin reversed CSCs to a pre-CSC-like stage and reduced the growth of human MLL leukemic cells [48]. CSCs rely on Wnt/β-catenin pathway in addition to other important pathways like PI3K/Akt/mTOR and JAK/STAT for acquiring therapy resistance [49]. In chronic myelogenous leukemia, deletion of β-catenin synergized with imatinib resulted in a delay of disease recurrence after imatinib discontinuation [50]. Wnt pathway has been shown to be upregulated in mantle cell lymphoma-initiating cells (MCL-IL) [51]. In addition, MCL-ILs were shown to be sensitive when targeted downstream at β-catenin-mediated transcription complex with inhibitors such as CCT036477, iCRT14, and PKF118-310. β-catenin mRNA or protein expression was shown to be upregulated in the bone marrow aspirates of patients with myelofibrosis and in other Philadelphia-negative myeloproliferative neoplasms [52, 53]. The Wnt/β-catenin pathway is also involved in the pathogenesis of multiple myeloma (MM), with silencing of the pathway resulting in autophagy and apoptosis in MM cells [54]. Loss of the deubiquitinating enzyme CYLD has been shown to enhance MM aggressiveness via Wnt pathway activation [55].

## WNT inhibitors in clinical development

**Fig. 2:**
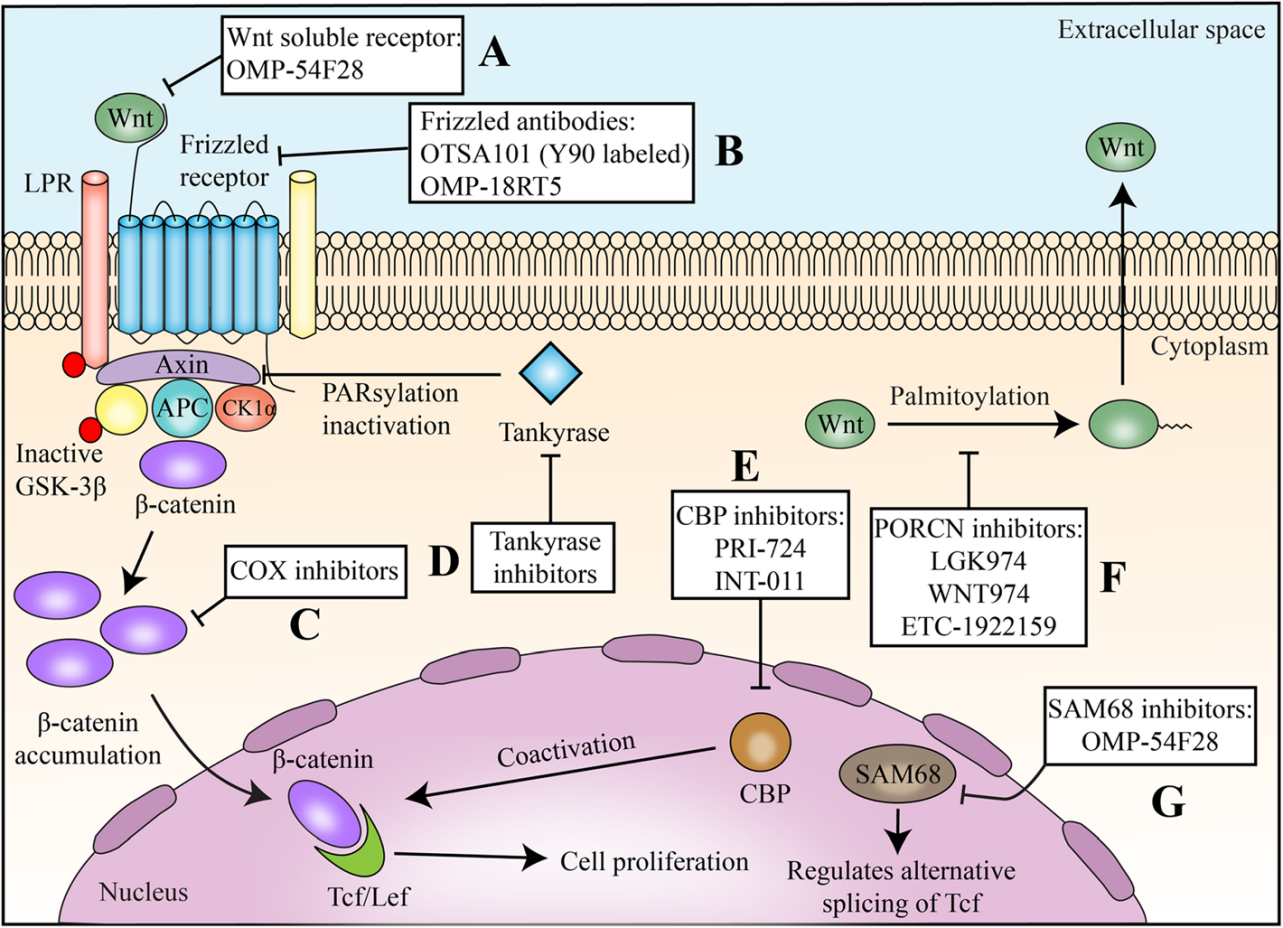
Therapeutic targets in Wnt/β-catenin pathway and developmental therapeutics. Multiple strategies have been under investigation to counteract the canonical pathway of Wnt signaling. **a**, **b** Wnt soluble receptors and antibodies directed to Frizzled receptors impair the interaction ligand/receptor and its conveyed signal. **c** COX inhibitors reduces β-catenin cytoplasmic levels through different ways. PGE2, the main product of COX2 enzyme, is thought to mediate β-catenin transcription. Also, COX inhibitors such as aspirin were related to increase β-catenin ubiquitination and proteasomal destruction. **d** Tankyrase activates axin through induction of PARsylation and proteasomal degradation; tankyrase inhibitors increase the levels of axin, facilitating the formation of the β-catenin destruction complex and reducing β-catenin availability. **e** CBP inhibitors reduce the interaction between CBP and Tcf/Lef, reducing Tcf/Lef activity. **f**. *PORCN* inhibitors reduce the essential palmitoylation of Wnt, precluding its release to the extracellular space. **g** SAM68 is a regulator of alternative splicing of Tcf and impairs β-catenin/Tcf/Lef interaction

Several therapeutic strategies have been developed with the aim to inhibit WNT-pathway, and many agents are undergoing early phase clinical trials (Table 1 and Fig. 2). Membrane bound *O*-acyl transferase porcupine (PORCN) has emerged as a molecular target of interest to treat WNT-driven cancers [56]. Addition of palmitoyl groups to WNT proteins is catalyzed by PORCN, a biochemical process known as palmitoylation or S-acylation in the endoplasmic reticulum, which enhances WNT secretion into the cytoplasm [57]. *WNT974*, a PORCN inhibitor, produced cytostatic effects in ovarian cancer cells in vitro [58] and decreased tumor growth and metastatic spread in head and neck squamous cell carcinoma models in vivo [59]. A phase I/II trial evaluating *WNT974* in combination with *LGX818*, a specific BRAF inhibitor, and cetuximab in patients with metastatic colorectal cancer bearing WNT and BRAF mutations is ongoing (NCT02278133). Another trial is evaluating WNT974 in patients with metastatic head and neck squamous cell carcinoma (NCT02649530).

**Table 1:** Therapeutic strategies against Wnt/beta-catenin in current clinical development

Mechanism of action	Agent Company name	Stage of clinical development	Status	Identifier	Details
PORCN inhibitor (blocks the secretion of Wnt ligands) PORCN inhibitor	WNT974 Array Biopharma	Phase 1	Active, not recruiting	NCT02278133	In combination with LGX818 and cetuximab; patients with BRAF-mut mCRC and WNT pathway mutations
		Phase 2	Withdrawn	NCT02649530	Patients with metastatic HNSCC; single-arm, non-randomized
	LGK974 Novartis	Phase 1	Recruiting	NCT01351103	Documented BRAF mut for mCRC and pancreatic cancer; tumors of any histological origin with documented genetic alterations upstream in the Wnt signaling; trial was suspended due to unknown reasons
	ETC-1922159	Phase 1a/1b	Recruiting	NCT02521844	Locally advanced or metastatic solid tumors
WNT-5a mimetic	Foxy-5 Wnt Research AB	Phase 1	Completed	NCT02020291	Metastatic breast, mCRC, or prostate cancer with loss of or reduced Wnt5a protein expression in IHC analysis
		Phase 1	Recruiting	NCT02655952	Metastatic breast, mCRC, or prostate cancer with loss of or reduced Wnt5a protein expression in IHC analysis
Sam68 modulator: interferes with the alternative splicing of Tcf	CWP232291 JW Pharmaceutical	Phase 1	Completed	NCT01398462	Relapsed or refractory AML, CMML, MDS, or high-risk myelofibrosis
		Phase1a/1b	Recruiting	NCT02426723	Relapsed or refractory MM
Wnt inhibitor	CGX1321 Curegenix Inc.	Phase 1	Recruiting	NCT02675946	Locally advanced or metastatic solid tumors
Inhibition of β-catenin recruitment through antagonizing its coactivator CBP (the binding protein of cAMP response element-binding protein CREB)	PRI-724 Prism Pharma Co.	Phase 1b	Completed	NCT01764477	Advanced or metastatic pancreatic adenocarcinoma, in combination with gemcitabine in the second line of treatment
		Phase 1/2	Active, not recruiting	NCT01606579	Advanced myeloid malignancies
		Randomized phase 2	Withdrawn	NCT02413853	Advanced mCRC, in combination with mFOLFOX6 + bevacizumab, in the first line of treatment
		Phase 1a/1b	Terminated	NCT01302405	Phase 1a: any advanced neoplasm Phase 1b: only patients with mCRC
Humanized monoclonal antibody OTSA101 against FZD10 and labeled with Y^90^	OTSA101 OncoTherapy Science	Phase 1	Active, not recruiting	NCT01469975	In patients with doxorubicin and ifosfamide-refractory synovial sarcoma
Monoclonal antibody against frizzled receptors	OMP-18R5 (Vantictumab) OncoMed Pharmaceuticals	Phase1	Recruiting	NCT01973309	In patients with metastatic breast cancer in combination with paclitaxel
Humanized monoclonal antibody (Mab) with neutralizing activity against Dkk-1	DKN-01 Leap Therapeutics, Inc.	Phase 1	Recruiting	NCT02013154	In combination with paclitaxel in esophageal neoplasms, adenocarcinoma of the gastroesophageal junction, gastroesophageal cancer, squamous cell carcinoma, and gastric adenocarcinoma
		Phase 1	Recruiting	NCT02375880	In combination with gemcitabine and Cisplatin in carcinoma of intrahepatic and extra-hepatic biliary systems, carcinoma of gallbladder, bile duct cancer, and cholangiocarcinoma
Antagonizes Wnt signaling through competes with the membrane-bound Fzd8 (decoy receptor)	OMP-54F28 (ipafricept) OncoMed Pharmaceuticals	Phase 1b	Active, not recruiting	NCT02069145	In patients with locally advanced or metastatic hepatocellular cancer, in combination with sorafenib
		Phase 1b	Recruiting	NCT02092363	In patients with recurrent platinum-sensitive ovarian cancer, in combination with paclitaxel and carboplatin
		Phase 1b	Active, not recruiting	NCT02050178	In patients with untreated stage IV metastatic pancreatic cancer, in combination with gemcitabine and nab-paclitaxel
		Phase 1	Completed	NCT01608867	Metastatic and unresectable refractory solid tumors

*Abbreviations*: *mCRC* metastatic colorectal cancer, *AML* acute myeloid leukemia, *CMML* chronic myelomonocytic leukemia, *MM* multiple myeloma, *Dkk1* Dickkopf-1, *Y90* radioactive yttrium^90^

WNT agonists, specifically WNT-5a activation, have shown to inhibit metastases. Increased WNT-5a signaling suppressed endothelial tumor cell migration and invasion and inhibited metastasis in model of breast cancer in vivo. *Foxy-5* is a WNT-5a mimic hexapeptide that binds and activates the WNT-5a receptors, Frizzled-2 and Frizzled-5 [60]. Two phase I trials are evaluating Foxy-5 in advanced solid tumors (breast, colorectal, and prostate) with loss or reduced expression of WNT-5a protein (NCT02020291 and NCT02655952).

*CWP232291* is a novel small molecule that binds Src associated with mitosis 68K protein (Sam68). Preclinical data showed selective inhibitory activity on a WNT gene reporter and decreased expression of the β-catenin target genes, cyclin D1 and survivin. CWP232291 is being tested in phase I clinical trials in patients with acute myeloid leukemia (NCT01398462) and relapsed or refractory myeloma (NCT02426723) [61].

*Genistein*, a dietary compound present in soy-based foods, is thought to mediate anticancer activity through pleiotropic mechanisms that may include the inhibition of WNT pathway [62]. Phase I and II studies investigating the clinical activity of genistein and other soy isoflavones compounds in cancer treatment and chemoprevention have been published with disappointing results [63–65].

*OMP-54F28* (*Ipafricept*) is a fusion protein that combines the immunoglobulin Fc domain with the cysteine-rich domain of frizzled family receptor 8 (Fzd8) competing with the native Fzd8 receptor for its ligands and antagonizes WNT signaling [66]. A dose-escalation phase I showed that OMP-54F28 was well tolerated and dose escalation cohorts with standard therapy in advanced hepatocellular cancers (NCT02069145), pancreatic cancers (NCT02050178), and recurrent platinum-sensitive ovarian cancer (NCT02092363) are under way [67]. Another monoclonal antibody against frizzled receptors OMP-18R5 is in phase 1 clinical trials for patients with metastatic breast cancer in combination with paclitaxel. DKN-01 is a humanized monoclonal antibody (Mab) with neutralizing activity against Dickkopf-1 (Dkk-1). Strong preclinical evidence of synergistic activity with chemotherapy agents is the basis for clinical testing of these agents in combination.

Preclinical studies have addressed several other strategies to counteract Wnt/β-catenin-mediated carcinogenesis, although they have not been yet tested in clinical trials. Inducing or stabilizing the “destruction complex” of β-catenin, therefore reducing its intracellular levels and precluding its transcriptional activity, is a promising strategy. Axin is a concentration-limiting component of the β-catenin destruction complex, and its stability is regulated by tankyrase, the key regulator enzyme that is responsible for poly(ADP-ribosyl)ation (PARsylation) of axin and induces its proteasomal degradation [68, 69]. Waaler et al. demonstrated the antitumoral effect of inhibiting tankyrase in a model of colorectal adenoma and adenocarcinoma [70]. Potential adverse events of this therapy include diarrhea and intestinal toxicity [71]. Tankyrase inhibition has been shown to revert resistance to PI3K and AKT inhibitors in colorectal cancer patient-derived sphere cultures and mouse tumor xenografts [72]. High nuclear β-catenin expression predicted resistance to PI3K and AKT inhibitors. Combined treatment with a WNT/tankyrase inhibitor reduced nuclear β-catenin, reverted resistance to PI3K and AKT inhibitors, and repressed tumor growth.

Another possible strategy to reduce signaling through the canonical pathway of Wnt/β-catenin consists of antagonizing β-catenin/TCF-mediated transcription. Because transcriptional regulation of the β-catenin/TCF complex needs some coactivators, such as the cAMP response element-binding protein (CREB)-binding protein (CBP), this molecule was considered a potential target for inhibition [73]. *INT-001*, a specific antagonist of CBP, showed antitumoral activity in preclinical models of pancreatic, colon, and tamoxifen-resistant breast cancers [74–76].

Multiple observational studies and randomized controlled trials have demonstrated a chemo-preventative role for aspirin, particularly in the development of colorectal neoplasia. Given the critical importance of WNT dysregulation in colorectal carcinogenesis, the interplay between aspirin and canonical WNT signaling has become a focus of investigation [77]. Through inhibiting cyclooxygenase-2 (COX-2), aspirin decreases the availability of prostaglandin E2 (PGE2), therefore reducing WNT levels [78]. Interestingly, cancer cells when exposed to aspirin in vitro led to the inhibition of WNT pathway through increasing β-catenin ubiquitination and consequent degradation, independent on COX inhibition as well [79]. Figure 2 summarizes the developmental strategies under investigation in Wnt/β-catenin pathway.

## Cancer immunity and Wnt/β-catenin pathway

Overexpression of immune checkpoint molecules in the tumor microenvironment has a critical role in antitumor immunity evasion and cancer progression [80]. Currently, four immune checkpoint inhibitors (ICI) are approved for treatment of cancer, including ipilimumab (anti-cytotoxic T-lymphocyte-associated protein 4 (anti-CTLA4)), nivolumab, pembrolizumab (anti-programmed death-1 (anti-PD-1)), and atezolizumab (anti-programmed death ligand-1 (anti-PD-L1)). Anti-PD-1/PD-L1 antibodies have demonstrated clinical activity in more than 15 cancer types, but the majority of patients with advanced cancer still do not derive clinical benefit from these drugs suggesting that immunosuppressive mechanisms within the tumor microenvironment may play a role in de novo resistance to these therapies.

Clinical responses to multiple T cell-based therapies, including immune checkpoint inhibitors, have correlated with tumors with a T cell-inflamed microenvironment [81, 82]. These tumors are characterized by infiltration of CD8^+^ T cells, chemokines, and an interferon signature, which ultimately correlate with improved survival and responses to immunotherapies [83, 84]. On the other hand, it has been recognized that tumors without T cell infiltration have worse prognosis and do not benefit from immunotherapy [84]. Thus, understanding mechanisms driving T cell exclusion is critical to increase the number of patients that will benefit from immunotherapy.

**Fig. 3:**
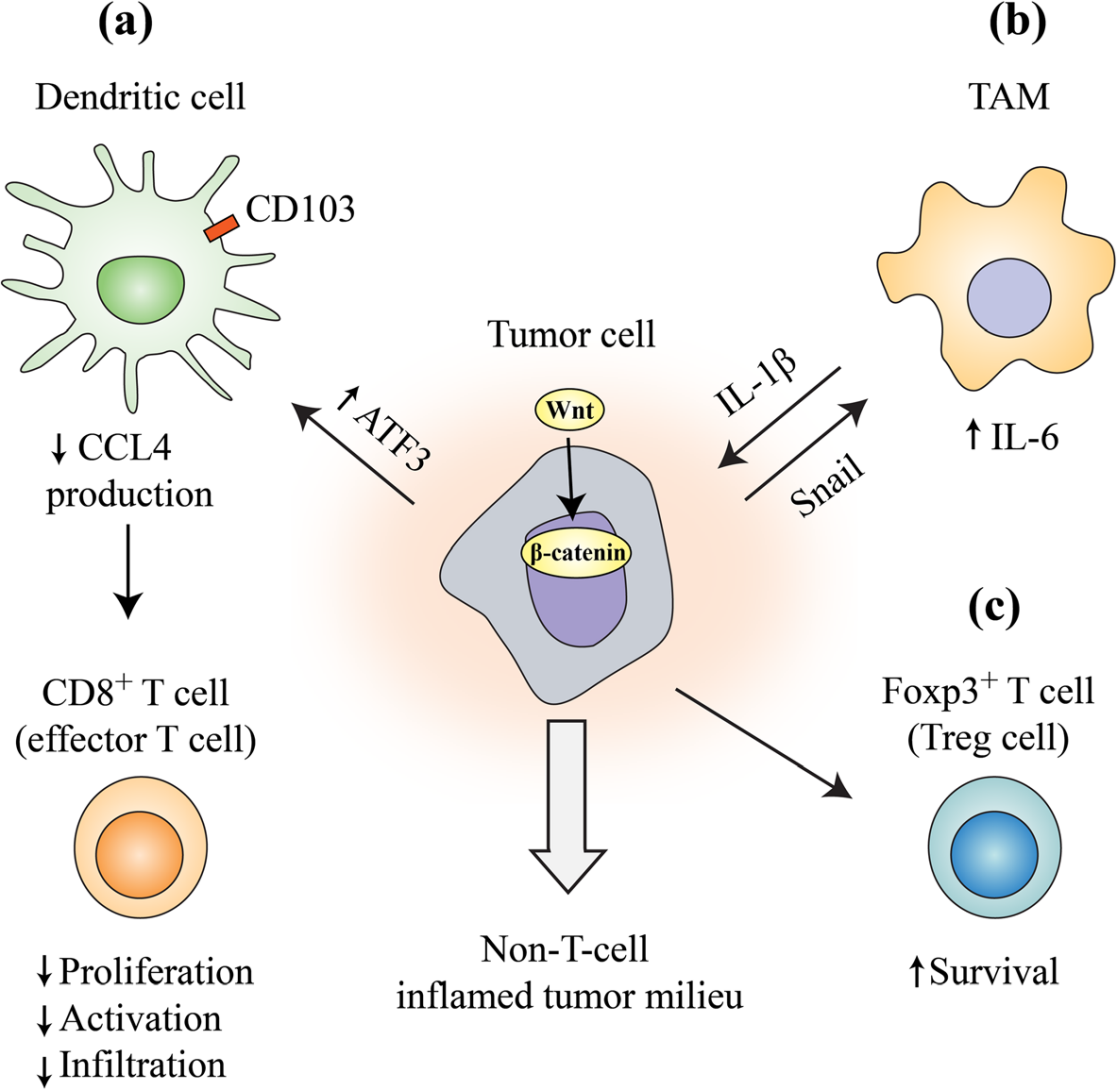
Mechanisms of immune exclusion through Wnt/beta-catenin pathway: Activation of Wnt/beta-catenin pathway in tumor leads to noninflammatory milieu through multiple mechanisms. **a** By acting on Batf3-lineage CD103^+^ dendritic cells, decreasing CCL4 production by inducing the gene expression of the transcription repressor ATF3. This in turn reduces CD8^+^ T cell priming and infiltration. **b** By interacting with tumor-associated macrophages (TAM) through Snail (a soluble factor product of a Wnt-regulated gene) which can in turn increase beta-catenin activity by IL-1β. **c** β-catenin can enhance Treg survival through unknown mechanisms

Wnt/β-catenin pathway has been identified as one of the important oncogenic pathway signaling related to immune evasion (Fig. 3) [85, 86]. In a teratoma model, enhanced expression of Wnt correlated with impaired immune cell recruitment. Both T- and B cell infiltration was reduced which was independent of teratoma size and differentiation suggesting impaired immune surveillance [87]. Luke et al. evaluated gene expression data from The Cancer Genome Atlas (TCGA) to segregate tumors based on a T cell-inflamed gene expression signature and classified 8890 tumor samples into T cell-inflamed, non-T cell inflamed, and intermediate subtypes [88]. Approximately one third of the interrogated tumors were characterized as non-T cell inflamed. They further interrogated exomic sequencing to address the presence of mutations in Wnt/β-catenin pathway in this subtype. Activating mutations in *CTNNB1* and inactivating mutations in negative regulators such as *Axin1*, *Axin2*, *APC1*, and *APC2* were related to non-T cell inflamed gene signature, accounting for about 13% of the sequenced tumors. Most of the mutations were noted in Exon 3 of the *CTNNB1* gene, with mis-sense mutation being the most common. Evidence of β-catenin pathway activation, in the presence of mutation or otherwise, was noted in 24 of 30 solid tumors of TCGA. Evidence for pathway activation without mutation was evidenced by increased expression of β-catenin pathway elements such as WNT ligands, Fzd receptors, or β-catenin itself. Immunohistochemistry demonstrated inverse correlations between β-catenin and CD8^+^ T cell infiltration.

These results suggest that increased expression of β-catenin in the tumor should be investigated and validated as a predictive tool to improve the selection of candidate patients for checkpoint inhibition therapy. In addition, one might hypothesize that inhibition of Wnt/β-catenin signaling could improve CD8^+^ T cell infiltration and priming, therefore possibly producing a more favorable scenario to immune checkpoint inhibition [89]. Thus, the investigation of the possible role of Wnt/β-catenin inhibitors as possible adjuvants to anti-PD1, anti-CTLA4, or anti-PD-L1 is an interesting strategy and should be evaluated in preclinical and clinical studies.

While tumor infiltration with CD8^+^ effector T cell is favorable, presence of regulatory T cells (Treg) has been associated with poor antitumor immunity. Tregs are known to suppress adaptive responses mainly by reducing CD8^+^ T cell proliferation, activation, and effector function [90, 91]. Some studies have shown that expression of β-catenin has been associated with Treg infiltration, survival, and activity [92, 93]. In an adoptive transfer mouse colorectal cancer model, β-catenin expression was enforced in intratumoral CD4^+^ T cells using lentiviral transduction. This led to increased IL-17a expression, enhanced proliferation, and inhibited apoptosis of colorectal cancer cells [87]. Active WNT signaling has been shown to disrupt Foxp3 transcriptional activity, a crucial step for both the development and function of regulatory T cells [94]. Hence, it has to be recognized that blocking WNT for immunomodulation is still controversial at this time. On the other hand, induction of Wnt signaling has shown to be important in maintenance of stemness of memory CD8^+^ T cells by blocking T cell differentiation [95]. This strategy may be used in generating memory T cell populations, thereby improving effectiveness of cancer vaccines, and may find applications in programming antitumor T cells for adoptive immunotherapy [96].

Wnt/β-catenin pathway has also been associated with modulation of innate immunity, such as dendritic cells [97]. Spranger et al. detected a correlation between activation of the Wnt/β-catenin signaling pathway and absence of a T cell gene expression signature in human metastatic melanoma [98]. Active β-catenin signaling was observed in 48% of non-T cell-infiltrated melanomas, which was thought to be the important pathway mediating immune exclusion. In mouse models, β-catenin induced expression of the transcription repressor ATF3, which suppressed the transcription of *CCL4*. The defective production of CCL4 led to impaired infiltration and activation of Batf3-lineage CD103^+^ dendritic cells, reduced CD8^+^ T cell priming and infiltration, and consequent non-response to immune check point blockade. In the absence of active β-catenin signaling, the normal production of CCL4 was restored, leading to CD103^+^ dendritic cell activation and infiltration and proficient priming of CD8^+^ T cell. Wnt/β-catenin signaling in intestinal dendritic cells has shown to regulate the balance between inflammatory versus regulatory responses in the gut [99]. β-catenin when expressed in intestinal dendritic cells was associated with expression of anti-inflammatory mediators such as retinoic acid-metabolizing enzymes, interleukin-10, and transforming growth factor-β, and the stimulation of Treg induction [100].

Wnt/Beta-catenin signaling has been shown to be involved in cross talk between cancer cells and tumor-associated macrophages. Kaler et al. demonstrated a possible role of interleukin-1β secreted by TAMs in the increased availability of β-catenin through phosphorylation of GSK3β in colon cancer cells, disrupting the function of β-catenin destruction complex [101]. Colorectal cancer cells stimulate macrophage production of IL-1β through Snail, a soluble factor product of a Wnt-regulated gene. These data demonstrated a novel Wnt-dependent cross talk mechanism between tumor cells and macrophages [102].

## Conclusions

The Wnt/β-catenin pathway is increasingly recognized as a potentially important target for anticancer therapies, with several relevant inhibitors at various stages of clinical development. The expanding role of immunotherapies in cancer and recent insight into the role of Wnt-pathway in cancer-related immune-regulation may give a new dimension to this field of developmental therapeutics. Only tumors with upregulated Wnt/β-catenin signaling such as colorectal cancer have been explored as targets for Wnt inhibition. However, with its role in immunomodulation, Wnt inhibitors may have a broader role in cancers such as melanoma, lung, and renal cancers where immunotherapy has come to the forefront. There are some nuances to be addressed in the exact role of Wnt signaling in immunomodulation before this could be adopted in clinical practice.

## Abbreviations

anti-CTLA4: Anti-cytotoxic T-lymphocyte-associated protein 4
anti-PD-1: Anti-programmed death-1
anti-PD-L1: Anti-programmed death ligand-1
APC: Adenomatous polyposis coli
CBP: cAMP response element-binding (CREB)-binding protein
COX-2: Cyclooxygenase-2
CRC: Colorectal cancer
CSC: Cancer stem cells
FGF: Fibroblast growth factor
Fzd8: Frizzled family receptor 8
GBM: Glioblastoma multiforme
MGMT: O6-methylguanine-DNA methyltransferase
MLL: Mixed-lineage leukemia
MM: Multiple myeloma
MMTV: Mouse mammary tumor virus
PARsylation: Poly(adenosine diphosphate-ribosyl)ation
PGE2: Prostaglandin E2
PORCN: *O*-acyl transferase porcupine
RCC: Renal cell carcinoma
TAM: Tumor-associated macrophage
TCGA: The Cancer Genome Atlas
Treg: Regulatory T cells
